# Estrogen-Like Properties of Fluorotelomer Alcohols as Revealed by MCF-7 Breast Cancer Cell Proliferation

**DOI:** 10.1289/ehp.8149

**Published:** 2005-09-01

**Authors:** Marleen Maras, Caroline Vanparys, Frederik Muylle, Johan Robbens, Urs Berger, Jonathan L. Barber, Ronny Blust, Wim De Coen

**Affiliations:** 1 Laboratory for Ecophysiology, Biochemistry, and Toxicology, University of Antwerp, Antwerp, Belgium; 2 Norwegian Institute for Air Research, Polar Environmental Centre, Tromsø, Norway; 3 Environmental Science Department, Lancaster University, Lancaster, United Kingdom

**Keywords:** cell cycle, E-screen, fluorotelomer alcohols, real-time PCR, xenoestrogen

## Abstract

We investigated estrogen-like properties of five perfluorinated compounds using a combination of three *in vitro* assays. By means of an E-screen assay, we detected the proliferation-promoting capacity of the fluorotelomer alcohols 1H,1H,2H,2H-perfluorooctan-1-ol (6:2 FTOH) and 1H,1H,2H,2H-perfluoro-decan-1-ol (8:2 FTOH). The more widely environmentally distributed compounds perfluoro-1-octane sulfonate, perfluorooctanoic acid, and perfluorononanoic acid did not seem to possess this hormone-dependent proliferation capacity. We investigated cell cycle dynamics using flow cytometric analyses of the DNA content of the nuclei of MCF-7 breast cancer cells. Exposure to both fluorotelomer alcohols stimulated resting MCF-7 cells to reenter the synthesis phase (S-phase) of the cell cycle. After only 24 hr of treatment, we observed significant increases in the percentage of cells in the S-phase. In order to further investigate the resemblance of the newly detected xenoestrogens to the reference compound 17β-estradiol (E_2_), gene expression of a number of estrogen-responsive genes was analyzed by real-time polymerase chain reaction. With E_2_, as well as 4-nonylphenol and the fluorotelomer alcohols, we observed up-regulation of trefoil factor 1, progesterone receptor, and *PDZK1* and down-regulation of *ERBB2* gene expression. We observed small but relevant up-regulation of the estrogen receptor as a consequence of exposures to 6:2 FTOH or 8:2 FTOH. The latter finding suggests an alternative mode of action of the fluorotelomer alcohols compared with that of E_2_. This study clearly underlines the need for future *in vivo* testing for specific endocrine-related end points.

Over past decades, a whole range of fluorinated chemicals have been synthesized and used as wetting agents, lubricants, corrosion inhibitors, insecticides, cosmetics, fire retardants, paper coatings, and surfactants (Renner 2001, 2003). The high stability of the carbon–fluorine bond and the inert characteristics of most of these compounds are regarded as attractive properties during the manufacture of plastics, electronics, textiles, or construction materials. For a long time, these fluorinated chemicals were considered metabolically inert and nontoxic (Sargent and Seffl 1970). However, environmental monitoring has shown that degradation to persistent molecules does happen on a large scale, as deduced from the worldwide distribution of compounds such as perfluorooctane sulfonate (PFOS), perfluorooctanoic acid (PFOA), perfluorohexanesulfonate, and perfluorooctanesulfonamide (Dimitrov et al. 2004; Dinglasan et al. 2004; Giesy et al. 2001; Hoff et al. 2003a, 2003b; Martin et al. 2004). Martin et al. (2004), for instance, describe fluorotelomer alcohols as potential sources of perfluorinated acids in regions as remote as the Arctic. Although the fluorotelomer alcohols are known as volatile chemicals that are capable of long-range atmospheric transport, biologic transformation seems to be the major degradation pathway causing deposition of mentioned perfluorinated acids. In addition, during past years many of the perfluorinated compounds have been found to possess undesirable toxic characteristics. As reviewed by Lau et al. (2004), perfluoroalkyl acids and their derivatives can cause developmental toxicity. Exposures of rats to PFOA may cause significant lags of weight gain of the offspring and a statistically significant increase in mortality in both male and female pups. PFOS exposure may provoke weight loss, hepatotoxicity, and reduction of serum cholesterol and thyroid hormones. PFOS apparently is also able to affect the neuroendocrine system (Austin et al. 2003). Female rats injected with PFOS have a disturbed estrous cyclicity and increased serum corticosterone levels with decreasing serum leptin levels. Increased norepinephrine concentrations were found in the paraventricular nucleus of the hypothalamus. The fact that perfluorinated chemicals may disturb the endocrine system is worrying and deserves further investigation. It is generally known that a well-functioning endocrine system depends on a delicate balance of hormones and hormone receptors that interact to provoke complex cellular signaling. Different environmental pollutants act as hormone mimics, binding to specific hormone receptors or indirectly interfering with hormone signaling. The consequence may be irreversible damage to the reproductive system, especially when living organisms are exposed during the embryonic stages of life (Degen and Bolt 2000; Rosselli et al. 2000). Behavioral changes are another well-known adverse effect of disturbance caused by endocrine-disruptive chemicals (Schantz and Widholm 2001). Although disturbance of the thyroid system seems to be provoked by specific perfluorinated chemicals such as PFOS, their potential for estrogen-like properties has not been reported until now. In the present study, we evaluated the capacity of perfluorinated compounds to reinduce cell proliferation of growth-arrested MCF-7 breast cancer cells. Using a combination of the E-screen assay, cell cycle analysis, and gene expression analysis of estrogen-responsive bio-marker genes, we demonstrate the estrogen-like properties of the fluorotelomer alcohols 1H,1H,2H,2H-perfluorooctan-1-ol (6:2 FTOH) and 1H,1H,2H,2H-perfluorodecan-1-ol (8:2 FTOH) *in vitro.*

## Materials and Methods

### Chemicals.

PFOS (perfluoro-1-octane sulfonate, tetramethylammonium salt; 98%), 17β-estradiol (E_2_), 4-nonylphenol (4-NP), and PFOA (pentadecafluorooctanoic acid; 96%) were purchased from Sigma-Aldrich (Steinheim, Germany), and perfluorononanoic acid (PFNA; 97%) was provided by Avocado Research Chemicals (Lancashire, UK). We purchased 2,3,7,8-tetrachlorodibenzo-*p*-dioxin (TCDD) from LGC Promochem (Middlesex, UK) and Dulbecco’s minimal essential medium (DMEM) and fetal bovine serum (FBS) from Gibco BRL Life Technologies (Paisley, Scotland). 1H,1H,2H,2H-Perfluorooctan-1-ol (6:2 FTOH) and 1H,1H,2H,2H-perfluorodecan-1-ol (8:2 FTOH) were purchased from Interchim (Montlucon, France). We assessed the purity of the fluo-rotelomer alcohol standards by gas chromatography coupled to full-scan mass spectrometry in the electron impact (EI), negative chemical ionization (NCI), and positive chemical ionization (PCI) modes. No impurities could be detected in either EI or PCI mode. In NCI mode, several signals were observed. Retention times and full-scan mass spectra of these signals revealed that they were very closely related to the main signal. They were tentatively elucidated as branched isomers of the FTOHs.

### Cell culture.

MCF-7 human Caucasian breast adenocarcinoma cells (no. 86012803; European Collection of Cell Cultures, Salisbury, UK) were cultured in 25-cm^2^ Nunc cell culture flasks (Nunc, Roskilde, Denmark) in standard growth medium (DMEM; Gibco BRL; supplemented with 2 mM glutamine, 1% nonessential amino acids, 5% heat-inactivated FBS, and phenol red as an indicator of pH). Cells were maintained in a 37°C incubator under a 5% CO_2_ atmosphere over a maximum of 30 passages. Cells were grown to 80–90% confluency before splitting them into one-fifths.

### E-screen assay.

We tested the proliferation-inducing capacity of chemicals using the E-screen assay according to Payne et al. (2000), with minor modifications to the protocol. In short, cells were seeded in black 96-well microtiter plates with clear, flat bottoms (Nunc) at a density of 2,000 cells/well. Cells attached overnight, after which standard growth medium was replaced with phenol red–free medium containing 5% charcoal/dextran-stripped FBS (CSFBS). A previous wash step with phosphate-buffered saline (PBS) assured removal of all estrogenic compounds. Cells were then incubated for 72 hr to make them estrogen responsive. Exposures to estrogenic compounds or xenoestrogens were started by adding chemicals to the cells from so-called chemical plates. In the latter plates, 2-fold dilution series of the chemicals were prepared, followed by transferring 20–180 μL in the wells of the cell plates. To guarantee minimal interference with cell physiologic responses, the concentration of the solvent (DMSO) did not exceed 0.1%. Plates were covered with gas-permeable sealing tape and incubated at 37°C for 6 days. Proliferation of cells was assessed using the CyQuant assay (Molecular Probes, Invitrogen, Merelbeke, Belgium) (Jones et al. 2001).

### Cell cycle analysis.

We seeded MCF-7 cells in 25-cm^2^ Nunc cell culture flasks in standard growth medium at a density of 300,000 cells/flask. Cells attached overnight, after which the growth medium was replaced by phenol red–free DMEM containing CSFBS. After incubation in estrogen-free medium for 72 hr, cells were exposed to E_2_ or test compounds at concentrations corresponding to the highest observed effect during the E-screen assay. After 24 hr, cells were harvested by trypsinization and washed twice in PBS. Next, cell nuclei were isolated and stained with propidium iodide (PI) for 1 hr as described by Vindelov et al. (1983). We performed flow cytometric analysis of cell cycle distribution and apoptosis with an LSRII flow cytometer with a 488-nm argon-ion laser (Becton Dickinson, San Jose, CA, USA). PI fluorescence was collected at bandpass 575/26 nm(FL2, red fluorescence channel) in the linear mode. For each measurement, data from 10,000 single cell events were collected, whereas cell aggregates and doublets were gated out in the two-parameter histograms of pulse height to pulse width of PI fluorescence. We analyzed cell cycle histograms using ModFit LT 3.0 software (Variety Software House, Topsham, ME, USA).

### Gene expression analysis by reverse-transcription polymerase chain reaction (PCR).

MCF-7 cells were seeded and grown in estrogen-free medium in a manner analogous to that described above for flow cytometric analyses. After 48 hr exposure to E_2_ or test compounds, total RNA was extracted from the cell using the RNeasy kit (Qiagen GmbH, Hilden, Germany) according to manufacturer’s instructions. RNA quantity and quality were evaluated using a NanoDrop spectrophotometer (Nanodrop Technologies, Wilmington, DE, USA). First-strand cDNA was synthesized using the Fermentas first-strand cDNA synthesis kit (MBI Fermentas Life Sciences, St. Leon-Rot, Germany). In brief, 1 μg total RNA was incubated with 0.5 μg oligo(dT)_18_ primer and incubated at 70°C for 5 min to denature RNA. Next, 20 U recombinant ribonuclease inhibitor and 1 mM dNTP mix were added to the RNA in the following reaction buffer: 50 mM Tris-HCl, pH 8.3; 50 mM KCl; 4 mM MgCl_2_; and 10 mM dithiothreitol. cDNA synthesis was started by adding 40 U M-MuLV (Moloney murine leukemia virus) reverse transcriptase at 37°C for 1 hr. Reaction was stopped by inactivation of the reverse transcriptase at 70°C for 10 min. The final volume (20 μL) was adjusted to 100 μL.

We designed highly purified salt-free OliGold primers (Eurogentec, Seraing, Belgium) for the internal control gene hypoxanthine phosphoribosyltransferase 1 (*HPRT1*) and for target genes estrogen receptor-α (*ESR1*), progesterone receptor (*PGR*), PDZ domain containing 1 (*PDZK1)*, and erb-b2 erythroblastic leukemia viral oncogene homolog 2 (*ERBB2*) using Roche Lightcycler software (Roche Diagnostics Belgium, Vilvoorde, Belgium). The sequences of the primers were as follows: for *HPRT1* (GenBank accession no. NM-000194; GenBank 2005), 5′-TGACACTGGCAAAACAATGCA-3′ and 5′-GGTCCTTTTCACCAGCAAGCT-3′; for *ESR1* (GenBank accession no. NM-000125), 5′-CCATGGAATAGCTAGT-3′ and 5′-CAGTGGCCTAAATCAA-3′; for *PGR* (GenBank accession no. NM-000926). 5′-TGGTCCTTGGAGGTCG-3′ and 5′-GCCTCTCGCCTAGTTG-3′; for *PDZK1* (GenBank accession no. AF012281), 5′-AACCATGACTCGCACA-3′ and 5′-AGCCGTCTGCAATAGC-3′; for *ERBB2* (nuclear receptor ERBB2; GenBank accession no. AF094517), 5′-GACACCTACGGCAGAG-3′ and 5′-GTGGCATCCACTGGAC-3′. The sequences of the primers for trefoil factor 1 (*TFF1*; pS2; GenBank accession no. X00474) as derived from Bièche et al. (2001) were 5′-CATCGACGTCCCTCCAGAAGAG-3′ and 5′-CTCTGGGACTAATCACCGTGCTG-3′.

For the Lightcycler reaction, we prepared a master mix of the following components to the indicated end concentration: 9 μL water, 1 μL forward primer (0.5 μM), 1 μL reverse primer (0.5 μM), and 4 μL Lightcycler Fast Start DNA Master SYBR Green I reagent mixture (Roche). The Lightcycler glass capillaries were filled with 15 μL master mix, and 5 μL cDNA (50 ng reverse-transcribed total RNA) was added as the PCR template. We used the following experimental protocol: denaturation program (95°C for 5 min), amplification program (95°C for 5 sec, 58°C for 5 sec, 72°C for 13 sec, with a single fluorescence measurement at the end of DNA synthesis), melting curve program (55–95°C with a heating rate of 0.1°C/sec and a continuous fluorescence measurement), and finally a cooling step to 40°C. Expression changes of specific target genes were deduced from shifts of the crossing points for the target genes in exposed versus non-exposed cells and normalized by comparison with the internal control gene *HPRT1*. The “crossing point” is the point at which the fluorescence rises appreciably above the background fluorescence. The relative expression ratio of the gene under study normalized by the internal control *HPRT1* gene was calculated using REST software (version 2; Pfaffl et al. 2002). We used the pairwise fixed reallocation randomization test included in the REST software to test the significance of the derived results. Each chemical treatment was performed in triplicate, which provided three replicate samples for the reverse-transcriptase (RT)-PCR analyses. To check amplification of the correct PCR products, we performed analysis by 1.5% agarose gel electrophoresis.

## Results

### Stimulation of MCF-7 cell proliferation.

For this study, we adapted the E-screen assay to 96-well microtiter plate format in order to test broad concentration ranges of compounds for their proliferation-inducing capacity in growth-arrested breast cancer cells. In accordance with Payne et al. (2000), we incubated the MCF-7 cells for 72 hr in estrogen-free growth medium to induce the estrogen responsive.

**Figure 1:**
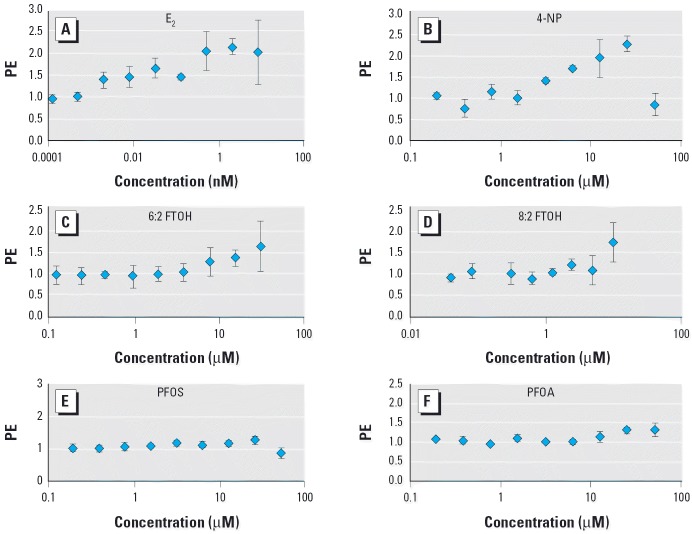
Analysis of estrogenicity of E_2_ (*A*), 4-NP (*B*), 6:2 FTOH (*C*), 8:2 FTOH (*D*), PFOS (*E*), and PFOA (*F* ) by the E-screen assay in MCF-7 cells. 0.1% DMSO was the solvent control. Results are expressed as mean ± SD of three replicates for each data point.

Figure 1 shows the proliferative effect (PE) as a result of exposure to a concentration range of the test compounds. PE (expressed as fold/control) is calculated as the ratio between the cell yield obtained with the test chemical and that with the hormone-free control. Table 1 presents the relative proliferative effect (RPE), which corresponds to the ratio of the maximal cell yield achieved by the minimum dose of xenobiotic relative to the reference compound (1 nM E_2_) and multiplied by 100. Table 1 also shows the relative proliferative potency (RPP), which corresponds to the ratio of the dose of E_2_ and that of xenobiotic needed to achieve a maximal PE. As demonstrated in Figure 1 and in Table 1, 6:2 FTOH and 8:2 FTOH behave like xenoestrogens *in vitro*. These compounds clearly induce cell proliferation at 10 μM, the concentration at which many other xenoestrogens are also active (Soto et al. 1995). Neither exposures to PFOS or PFOA within the concentration ranges applied nor the negative control TCDD led to increased cell proliferation.

### Effects of perfluorinated compounds on cell cycle distribution.

We performed further evaluation of the fluorinated compounds by flow cytometric analyses of the cell cycle. Although growth-arrested MCF-7 cells are predominantly in the G0/G1-phase of the cell cycle, addition of (xeno-)estrogens makes cells proliferate again, shown by the marked increase in the percentage of cells in the S-phase after 24 hr of exposure.

**Figure 2:**
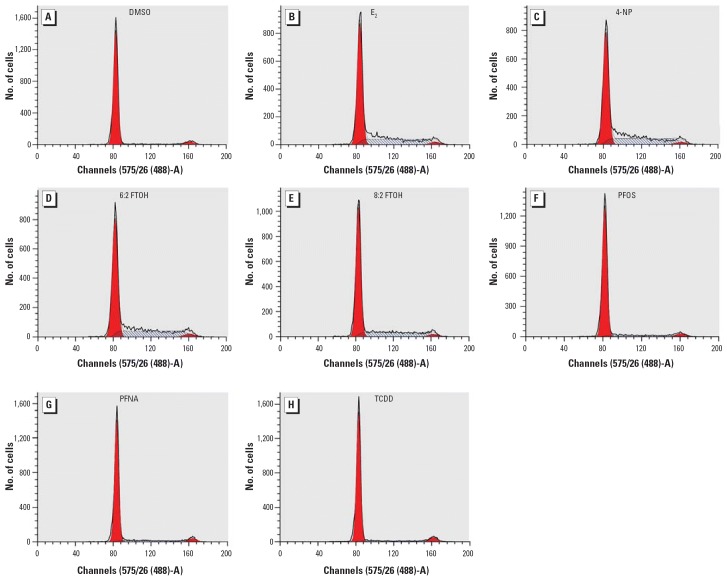
Histograms of DNA content showing the effects of perfluorinated compounds on cell cycle distribution. (*A*) 0.1% DMSO (solvent control). Cells were cultured in DMEM plus 5% CSFBS for 72 hr before exposing them to estrogenic compounds (*B*, 1 nM E_2_; *C*, 10 μM 4-NP; *D*, 30 μM 6:2 FTOH; and *E*, 10 μM 8:2 FTOH) and non-estrogenic perfluorinated compounds (*F*, 50 μM PFOS; *G*, 50 μM PFNA) for 24 hr. 4-NP (*C*) was the positive control, and 10 nM TCDD (*H*) was the negative control.

In Figure 2, the histograms of DNA content show increases in S-phase as the result of exposures to the fluorotelomer alcohols and 4-NP. In Table 2, results are expressed as percentages of cells in the different phases of the cell cycle. The increases of cell numbers in the S-phase range from 6% (solvent control) to almost 35% (1 nM E_2_ and 4-NP), approximately 31% (30 μM, 6:2 FTOH), and approximately 29% (10 μM, 8:2 FTOH). PFOS, PFOA, or PFNA, at concentrations ≤ 50 μM, did not affect proliferation. As shown in Table 3, fluorotelomer alcohols stimulate MCF-7 cells at concentrations ranging between 10^−6^ and 10^−5^ M. At 10^−7^ M, the stimulatory effect is lost. These results corroborate the findings from the E-screen assay and demonstrate the reinduction of cell proliferation of growth-arrested MCF-7 cells within a much shorter exposure period.

### Expression alterations of estrogen-responsive genes.

**Figure 3:**
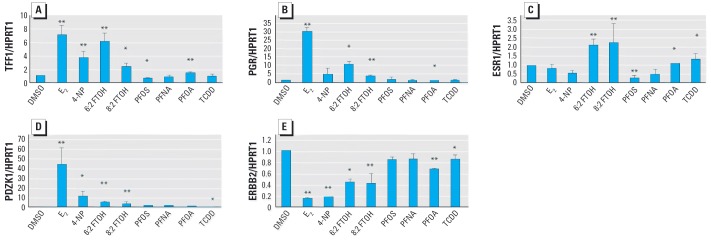
Effect of perfluorinated chemicals on mRNA expression of estrogen-responsive genes in MCF-7 cells were treated with 0.1% DMSO, 1 nM E_2_, 10 μM 4-NP, 30 μM 6:2 FTOH, 10 μM 8:2 FTOH, 50 μM PFOS, 50 μM PFNA, 50 μM PFOA, or 10 nM TCDD. After exposure to the test compounds for 48 hr, mRNA levels of *TFF1* (*A*), *PGR* (*B*), *ESR1* (*C*), *PDZK1* (*D*), and *ERBB2* (*E*) were measured by real-time PCR and normalized using *HPRT1* as an internal control. Results are means from three replicate measurements and are expressed as fold relative to 0.1% DMSO; error bars indicate SD. *p < 0.05. **p ≤ 0.001.

Reverse-transcription PCR was performed to analyze the expression of specific estrogen-responsive biomarker genes after 48 hr of exposure. As presented in Figure 3, significantly high up-regulation of *TFF1* mRNA was observed with E_2_ (7×), 4-NP (3.8×), 6:2 FTOH (6.2×), and 8:2 FTOH (2.4×). A small up-regulation was also observed with PFOA (1.4×), and a small but significant down-regulation was observed upon exposure to PFOS (1.7×). Exposure to E_2_ induced very high *PGR* mRNA levels (30×), whereas significant up-regulations were seen with 4-NP (4.5×), 6:2 FTOH (10.4×), and 8:2 FTOH (2.4×). *ESR1* was down-regulated upon exposures to E_2_ (1.33×) or 4-NP (2.1×). With 6:2 FTOH and 8:2 FTOH, however, small but significant up-regulations of *ESR1* were observed (2.2× up with both compounds). PFOS exposure resulted in a significant small down-regulation (3.8×). We studied the expression levels of two additional estrogen-responsive genes in order to further reveal the similarity of the telomeric alcohols to E_2_. An up-regulation of *PDZK1* expression was observed with E_2_ (41×), with 4-NP (13.2×) as well as with both perfluorinated telomeric alcohols (5.4× with 6:2 FTOH and 2.4× with 8:2 FTOH). Significant down-regulation of *ERBB2* was observed with E_2_ (4.5×) and 4-NP (4.4×), whereas less pronounced but nonetheless significant down-regulations were also observed with 6:2 (2.4×) FTOH and 8:2 FTOH (2.4×). A small down-regulation of *ERBB2* was observed with PFOA (1.5×).

## Discussion

A range of fluorinated chemicals synthesized during the past few years are promising for various industrial applications (Lehmler 2005). However, because of the persistent nature of these chemicals, monitoring their environmental fate and their ecotoxicologic characteristics is especially warranted (Van de Vijver et al. 2003, 2004). Chemicals that are difficult to degrade biologically may bioaccumulate and may affect the health of humans and biota. Disturbance of the endocrine system is one example of the toxic effects that need careful follow-up. Endocrine disruptors may mimic hormones or interfere indirectly with hormonal pathways. Damage caused by these compounds may, in the long term, lead to drastic effects such as decreased reproduction, or perhaps more subtle effects, such as a disturbance to the developmental system resulting in behavioral effects (e.g., learning disorders). Until now, studies that investigated endocrine-disrupting capacities of fluorinated compounds have been difficult to find. In a review describing developmental toxicity of perfluoroalkyl acids, Lau et al. (2004) highlighted the disturbances of the thyroid gland caused by such compounds (e.g., PFOS causing hypothyroxinemia). Because thyroid hormones are known to regulate brain development, these findings merit further research.

During the present study, the estrogen-like capacities of the fluorinated compounds 6:2 FTOH, 8:2 FTOH, PFOS, PFOA, and PFNA were studied *in vitro*. We used a combination of three different *in vitro* assays to demonstrate these findings. First, we used the E-screen assay, a commonly used high-throughput test to detect estrogen-like compounds in environmental samples. Using this assay, we found that 6:2 FTOH and 8:2 FTOH behave like xenoestrogens *in vitro*. These compounds clearly induce cell proliferation at 10 μM, the concentration at which many other xenoestrogens are also active (Soto et al. 1995). To complement and corroborate our E-screen results, we studied cell cycle dynamics using flow cytometry. Cells in estrogen-free growth medium do not enter the S-phase easily (Villalobos et al. 1995). Although > 80% of the MCF-7 cells were in growth arrest (G0/G1-phase of the cell cycle), the addition of xenoestrogens stimulated cells to synthesize new DNA in preparation of cell division, as revealed by the significant increase of the number of cells in the S-phase of the cell cycle. These increases were clearly observable after 24 hr exposure to the telomeric alcohols. Upon comparison of the E-screen assay with flow cytometric analyses, we found similar effective concentrations of E_2_, 4-NP, and the fluorotelomer alcohols. However, the estrogen-like compounds only induced 2- to 3-fold increases of cell numbers during the E-screen assay, whereas during flow cytometric analyses, we observed up to 5-fold increases of cells in S-phase. Because the exposure periods of E-screen (6 days) and flow cytometric analyses (24 hr) are very different, we also studied cell cycle dynamics after longer exposure periods. After 48 hr, we observed a significant drop of the percentage of cells in S-phase (results not shown). Apparently, cells that are boosted to reenter the cell cycle by a 24-hr xenoestrogen exposure rapidly return to a more modest proliferation rate after 48 hr. One possible explanation is based on the fact that MCF-7 cells express the estrogen receptor as well as the progesterone receptor. Cross-talk exists between nuclear receptors. For instance, progesterone receptor A may act as a repressor of transcriptional activities of different other members of the nuclear receptor family, among them the estrogen receptor (Kraus et al. 1995, 1997). A variation in parameters, such as the ratio of progesterone receptor A to progesterone receptor B, may be the consequence of exposures to (xeno-)estrogens, and apparently this altered ratio may dramatically affect estrogen receptor signaling activities. Another important issue to investigate further is the fact that nuclear receptor levels differ in different breast cancer cell lines and even within different clones of a cell line, which may explain why xenoestrogens provoke different cell proliferation responses with different MCF-7 cell lines (Coser et al. 2003; Villalobos et al. 1995).

In order to unravel the mode of action of estrogens and xenoestrogens, gene expression analysis of selected estrogen-responsive genes was performed (Frasor et al. 2003; Inoue et al. 2002). The expression changes of a small number of estradiol-responsive genes such as *TFF1*, *PGR*, *ESR1*, *PDZK1*, and *ERBB2* were studied using reverse-transcription PCR. *TFF1* is generally accepted as one of the most reliable estrogen-responsive biomarker genes for *in vitro* MCF-7 breast cancer cells (Jorgensen et al. 2000; Olsen et al. 2003; Wang and Lou 2004). This factor, also known as pS2, belongs to a family of “trefoil peptides” probably involved in the regulation of cell proliferation. *PDZK1* is another frequently reported estrogen-responsive gene (Ghosh et al. 2000; Yoshida et al. 2004). Proteins containing the PDZ domain are involved in organizing cell membrane proteins and are also involved in linking transmembrane proteins to the actin cytoskeleton (Yang et al. 1998). The induction of *PDZK1* by E_2_ is suggested to play a crucial role in membrane alterations that happen upon estrogen treatment such as formation of microvilli. *ERBB2* is a transmembrane tyrosine kinase receptor playing a role in mammary oncogenesis. This receptor is up-regulated in MCF-7 cells grown in estradiol-free medium and is down-regulated again upon addition of E_2_ (Martin et al. 2004; Vendrell et al. 2004). The estrogen-responsive genes *TFF1*, *PGR*, *PDZK1*, and *ERBB2* were commonly responsive to E_2_ as well as to the xenoestrogen 4-NP and the tested fluorotelomer alcohols. However, although a common up- or down-regulation is observed, the degree of response of the different genes may differ markedly, probably as a consequence of structural differences of the xenoestrogens (Terasaka et al. 2004). These differences may be responsible for and reflect the modes of action. Such differences were also found during the present study. For instance, although 4-NP appears to be a weaker inducer of *TFF1* and *PGR* than 6:2 FTOH, it seems to be a stronger inducer of *PDZK1*. To discriminate between different xenoestrogens with different modes of action, many more estrogen-responsive genes should be studied. Microarray analyses are used to characterize and classify known and newly detected xenoestrogens according to their different modes of action. The MCF-7 cell line may be an attractive model for this kind of study, due to possible cross-talk between the different hormone receptors of this cell line (Lange et al. 2005). Although we observed a down-regulation of *ESR1* expression by E_2_ and 4-NP, the fluorotelomer alcohols used in the present study caused a significant, approximately 2-fold up-regulation of this receptor. This finding suggests an alternative mode of action, different from that of the reference compound E_2_. Up-regulation of the estrogen receptor by presumed xenoestrogens is not unusual, as previously demonstrated for endosulfan, toxaphene, and dieldrin (Soto et al. 1995).

These results warrant further work toward *in vivo* testing for specific endocrine-disruptive end points. Our results have been generated with an *in vitro* system using a single cell line, confirming the estrogen-like properties at different molecular levels. However, at present, it is not at all clear whether fluorotelomer alcohols are causing endocrine disruption under realistic environmental exposure conditions. Information concerning *in vivo* studies is just becoming available. A one-generation reproductive toxicity study with rats suggests no harmful effect on reproduction (Mylchreest et al. 2005). These authors did not observe any test-substance–related effects on estrous cycle parameters or sperm morphology, motility, or epididymal sperm counts in the first parental generation. Mylchreest et al. (2005) detected no clear estrogen-like properties in this rat *in vivo* study. In another long-term rat exposure study (90 days) using a mixture of fluorotelomer alcohols (at doses ≥ 100 mg/kg/day), Ladics et al. (2005) found a persistent elevation of liver weights and thyroid follicular hypertrophy. One possible explanation for the observed discrepancy between our *in vitro* results and the few *in vivo* data might be related to differences in fluorotelomer metabolism between the breast cancer cell line and the *in vivo* exposure condition. These compounds may be converted in rats to other fluorinated molecules, such as PFOA, and hence, fluorotelomer alcohol exposures result in PFOA-like *in vivo* effects (Berger U, personal communication; Lehmler 2004). Clearly, these possible contradictions between *in vitro* screening assays and *in vivo* data merit further study. At present, it is questionable whether the fluorotelomer alcohols used in the present study might act as endocrine-disrupting xenoestrogens on various organisms that might have different metabolizing capacities. Organisms or individuals with a low fluorotelomer-metabolizing activity might be at risk.

Regarding environmental exposure conditions, few data are available at present. Although fluorotelomer alcohols have been detected in the atmosphere at concentrations up to 135 pg/m^3^ (Martin et al. 2004), there are presently no records of these compounds in surface water, sediment, or wildlife.

In conclusion, we characterized fluorotelomer alcohols as xenoestrogens *in vitro*. The structural similarities of these compounds and 4-NP, the reference xenoestrogen, offer a possible explanation why these new compounds may act as ligands for the estrogen receptor (Katzenellenbogen 1995). *para*-Alkylphenols have been shown to bind fully to the estrogen receptors in a dose-dependent manner, and the interaction of alkylphenols with the receptor became stronger with an increase in the number of alkyl carbons (Tabira et al. 1999). In the present study, 6:2 FTOH was characterized as a stronger xenoestrogen than 8:2 FTOH. It is very likely that the chain length of the alkyl group is the responsible factor. The characterization of fluorotelomer alcohols as *in vitro* xenoestrogens demonstrates the need to carefully monitor their environmental distribution and to further investigate the effects of perfluorinated compounds on biota.

## Figures and Tables

**Table 1:** Estrogenic effect of perfluorinated compounds according to the E-screen assay.

Compound	Concentration	RPE	RPP
E_2_	1 nM	100	1
4-NP	10 μM	100	10^−4^
6:2 FTOH	10 μM	50	10^−4^
8:2 FTOH	10 μM	46	10^−4^

**Table 2:** Results of cell cycle analyses of MCF-7 cells exposed to different perfluorinated compounds given as the percentage of cells by phase.

Treatment	G1/G0-phase	S-phase	G2/M-phase
0.1% DMSO	90.43 ± 1.01	6.00 ± 1.00	3.57 ± 1.64
E_2_ (1 nM)	63.14 ± 1.61	34.84 ± 2.48	2.03 ± 0.88
4-NP (10 μM)	63.71 ± 1.86	34.64 ± 0.47	1.64 ± 1.41
6:2 FTOH (30 μM)	66.98 ± 4.09	30.83 ± 3.23	2.19 ± 0.87
8:2 FTOH (10 μM)	68.53 ± 1.48	29.36 ± 1.78	2.11 ± 0.49
PFOS (50 μM)	85.63 ± 0.94	10.49 ± 0.71	3.87 ± 0.73
PFNA (50 μM)	85.53 ± 1.64	9.89 ± 1.53	4.57 ± 0.42
PFOA (50 μM)	83.57 ± 1.04	9.17 ± 0.57	6.83 ± 0.59
TCDD (10 nM)	87.46 ± 0.30	7.96 ± 0.37	4.58 ± 0.45

Values are mean ± SD of three measurements per treatment. During all measurements, coefficient of variation values of the G0/G1 peak were < 3.6 (*n* = 3).

**Table 3:** Results of cell cycle analyses of MCF-7 cells exposed to concentrations of fluorotelomer alcohols given as the percentage of cells by phase.

Treatment	G1/G0-phase	S-phase	G2/M-phase
0.1% DMSO	90.43 ± 1.01	6.00 ± 1.00	3.57 ± 1.64
6:2 FTOH (30 μM)	66.98 ± 4.09	30.83 ± 3.23	2.19 ± 0.87
6:2 FTOH (3 μM)	80.14 ± 3.04	15.63 ± 3.07	4.22 ± 0.34
6:2 FTOH (0.3 μM)	88.22 ± 0.48	7.89 ± 0.53	3.89 ± 0.38
8:2 FTOH (10 μM)	68.53 ± 1.48	29.36 ± 1.78	2.11 ± 0.49
8:2 FTOH (1 μM)	82.70 ± 1.04	12.72 ± 0.84	4.58 ± 0.35
8:2 FTOH (0.1 μM)	87.04 ± 0.47	8.41 ± 0.04	4.56 ± 0.43

Values are mean ± SD of three measurements per treatment. During all measurements, coefficient of variation values of the G0/G1 peak were < 3.6 (*n* = 3).
